# A Randomized Placebo-Controlled Study on the Effectiveness of the “Three Good Things for Others” Intervention

**DOI:** 10.3389/fpsyg.2021.661336

**Published:** 2021-05-19

**Authors:** Mariola Laguna, Michał Kȩdra, Zofia Mazur-Socha

**Affiliations:** Institute of Psychology, The John Paul II Catholic University of Lublin, Lublin, Poland

**Keywords:** prosocial behavior, affect, positive orientation, intervention, positive psychology, childhood memories, randomized controlled trial

## Abstract

The aim of our study was to test the effectiveness of the “three good things for others” intervention. We used the randomized controlled trial method, with four measurements (pretest, posttest, follow-up after 2 weeks, follow-up after 4 weeks) and with random assignment of participants to experimental and placebo control groups. We investigated the effects of the intervention on prosocial behavior, and in addition on positive and negative affect, and positive orientation (a general tendency to approach reality in a positive way). The results showed an increase in positive affect and a decrease in negative affect in the experimental group a day after the intervention. These effects, however, did not endure over the next 2 or 4 weeks. We also observed a statistically significant increase in prosocial behavior in the placebo control group, in which participants were engaged in a task of recalling childhood memories. The results are discussed and recommendations for future studies are proposed.

## Introduction

Prosocial behavior, defined as behavior undertaken voluntarily to benefit others, may include a range of actions such as helping, sharing, caring, comforting, volunteering, and donating (Eisenberg et al., 2007; Alessandri et al., 2009; Caprara et al., 2012). It is socially valued, increasing the quality of interactions between individuals and among groups (Eisenberg et al., 2007); it is highly significant for economic and societal outcomes, too (Meier, 2007). Therefore, evidence from psychological research is called for to propose and test interventions promoting prosocial behavior (e.g., Meier, 2007).

Responding to this call, we have developed a new intervention, called “three good things for others,” inspired by positive psychology interventions (Seligman et al., 2005; Gander et al., 2018). This paper presents a study testing its effectiveness.

A systematic review of studies on interventions promoting prosocial helping behavior (Laguna et al., 2020c) and a meta-analysis of interventions targeted at children and adolescents (Mesurado et al., 2019) demonstrated that some of these interventions were, at least moderately, effective. However, the existing research has focused mostly on children, and the interventions were typically delivered at schools and kindergartens. Most of them were relatively long (lasting even years) and involved face-to-face contact. Moreover, the authors of these reviews concluded that more studies were needed to strengthen the evidence base for the effectiveness of prosocial interventions. Therefore, we have developed a new intervention, which is targeted not at children but at young people, which is not a long-term program but short one, and which can be delivered online rather than in face-to-face contact.

Our intervention was inspired by positive psychology interventions, defined as “treatment methods or intentional activities that aim to cultivate positive feelings, behaviors, or cognitions” (Sin and Lyubomirsky, 2009, p. 468). They may also influence social functioning, for example increasing trust in social relations (Drazkowski et al., 2017). One of them, well-validated, is the “three good things” intervention (Seligman et al., 2005). Participants are asked to “write down three things that went well each day and their causes every night for one week” and to “provide a causal explanation for each good thing” (Seligman et al., 2005, p. 416). This intervention and its several variants, such as “three funny things” or “three pleasurable things,” are meant to facilitate pleasurable life and happiness (see Gander et al., 2018) and indeed demonstrated significant and sustainable effects (e.g., Bolier et al., 2013). As our aim in the “three good things for others” intervention was to stimulate prosocial behavior, every evening for 1 week we asked participants to think about and write down three good things that they wanted to do for other people the next day and then to perform these actions. This means our intervention differs in aim from positive interventions and encourages prosocial actions. Being short and focused on everyday experiences, it responds to the call to develop “wise interventions” (Walton, 2014).

Research demonstrated that it was possible, at least to some degree and for some time, to increase prosocial behavior through interventions (Mesurado et al., 2019; Laguna et al., 2020c). It was therefore reasonable to expect that our intervention would also result in the enhancement of prosocial behavior, encouraging it in different situations. We focused on altruistically motivated prosocial behavior reflected in social value orientation (Böckler et al., 2016, 2018). It is defined in terms of “the weights people assign to their own and others' outcomes in situations of interdependence” (Balliet et al., 2009, p. 533) and usually measured using the decomposed game (Van Lange et al., 1997; Balliet et al., 2009). Prosocial choices maximize outcomes both for a participant and for others and minimize differences between outcomes for themselves and for others (Van Lange et al., 1997). We expected that our intervention would increase prosocial behavior, including prosocial choices in the decomposed game. Therefore, we hypothesized that in the experimental group, as compared to the control group, the level of prosocial behavior would be higher after the intervention than before the intervention.

Theoretical premises and previous research results (Alessandri et al., 2009; Laguna and Alessandri, 2020; Laguna et al., 2020a) demonstrate that engagement in prosocial behavior may lead to higher positive affect, lower negative affect, and higher positive beliefs, which means the levels of these variables may also increase as a result of our intervention. We selected these variables to compare the effects of our intervention with effects of positive psychology interventions. We explain this in detail below.

Affect as consciously accessible feelings evident in moods and emotions (Fredrickson, 2001) is considered variable over time, and its valance is treated as a basic dimension, allowing to distinguish positive and negative affect (Watson et al., 1988). Empirical findings have demonstrated that affect, both positive and negative, changes as a result of goal realization and social relations (Carver, 2005, 2006; Plemmons and Weiss, 2013). Both experimental and longitudinal studies support the association between affect and prosocial behavior (for a review, see Moore et al., 2018) showing that the tendency to engage in prosocial acts predicted affective experiences (Laguna and Alessandri, 2020). Moreover, one of the important working mechanisms of positive interventions is the elicitation of positive affect (Gander et al., 2018), and they have significant and sustainable effects on subjective well-being (Sin and Lyubomirsky, 2009; Bolier et al., 2013; Hendriks et al., 2020). Similarly, prosocial interventions may operate by changing individuals' affective experiences (Donald et al., 2019), and in addition to increasing prosociality they may reduce depressive symptoms (Schonert-Reichl et al., 2015). We therefore expected that our intervention would increase the level of positive affect and decrease the level of negative affect in the experimental group, as compared with the control group.

Not only affect but also the beliefs a person holds (Carver, 2005; Caprara et al., 2012) may be related to prosocial behavior. Numerous studies has demonstrated that three intercorrelated positive beliefs: about oneself (self-esteem), about one's life (life satisfaction), and about the future (optimism), together form a general tendency to approach reality in a positive way, called positive orientation (for a review, see Caprara et al., 2019). Research shows that this cognitive orientation (and its three sub-components) is positively related to prosocial behavior (Thoits and Hewitt, 2001; Eisenberg et al., 2007; Laguna and Alessandri, 2020; Laguna et al., 2020a). Initially, positive orientation was treated as a relatively stable disposition, to some degree inherited (Caprara et al., 2009). Recently, however, attention has turned to its variability rather than stability (Caprara et al., 2019; Laguna, 2019). This view was supported by evidence from longitudinal studies (Alessandri et al., 2014) showing that tendency to engage in prosocial acts predicted the positive orientation (Laguna and Alessandri, 2020). Moreover, positive psychology interventions may work through the elicitation of positive thoughts (Gander et al., 2018). Taking these results into account, we predicted that our intervention may increase the level of positive orientation in the experimental group, as compared with the control group.

Based on recommendations concerning research methodology coming from reviews of studies on positive interventions (Bolier et al., 2013) and of interventions stimulating prosocial behavior (Laguna et al., 2020c), we applied a randomized controlled trial design with four measurements and random assignment of participants to experimental and placebo control groups. We used the “early memories” exercise (Seligman et al., 2005) as a placebo control condition, as it has been used in many previous intervention studies (e.g., Gander et al., 2013, 2018), including those testing the original “three good things” intervention (Seligman et al., 2005).

## Methods

### Procedure

University students were encouraged to participate in the project concerning personal development through social media and through invitations during academic classes and at dormitories. We informed students that financial prizes would be drawn among the participants who finished the project. Those who agreed to participate provided their e-mail addresses and consented to the processing their personal information. They were assigned to an experimental (152 students) or control group (151 students) through non-return randomization using SPSS software.

**Figure 1:**
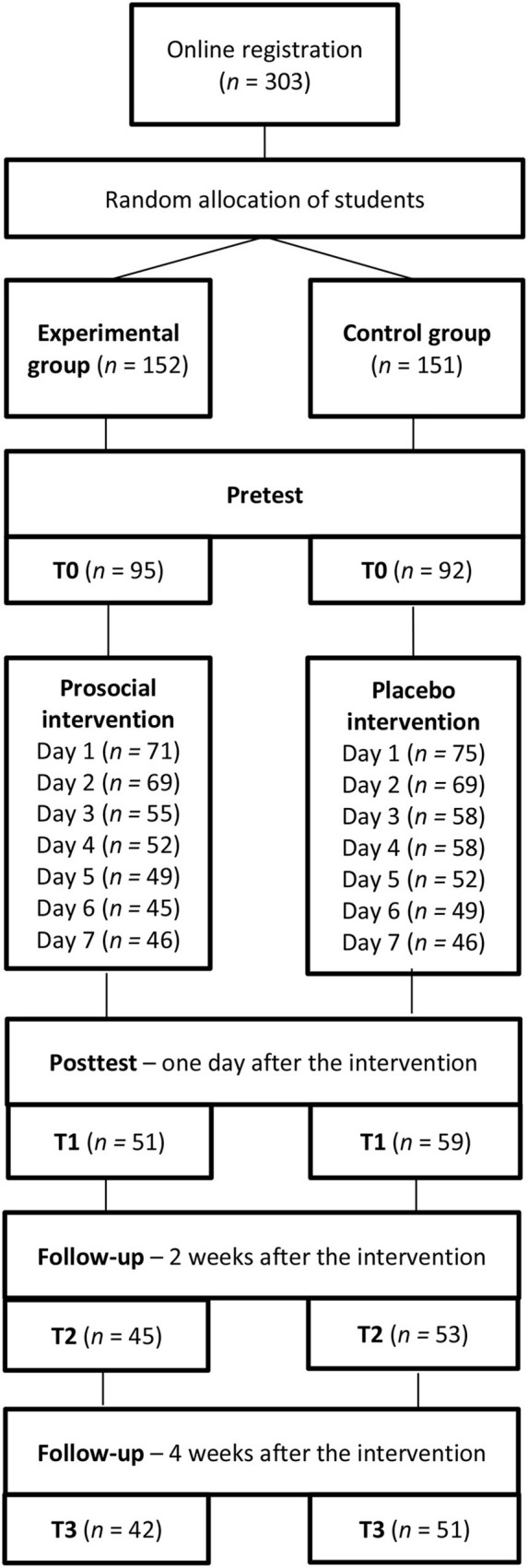
Flow of participants during the successive stages of the study.

Participants received all materials via email. They participated in the intervention or placebo control condition (Figure 1). They completed the measures online, immediately before the intervention (T0 pretest), a day after the intervention (T1 posttest), 2 weeks after the intervention (T2 follow-up), and 4 weeks after the intervention (T3 follow-up).

### Participants

Of the initially recruited 303 Polish students, 187 participated in the pretest (80.2% women). They were 18–29 years old (*M* = 21.43, *SD* = 2.07). The flow of participants is presented in Figure 1.

### Intervention

To provide comparable conditions for experimental and control groups, simultaneously with the intervention, the control group was engaged in placebo activities similar in terms of structure and timing. On the first day, students from both groups received a short presentation. Next, each evening for seven consecutive days they received daily tasks. Details are available from the first author.

#### “Three Good Things for Others” Intervention

Students received a presentation that explained helping others and provided examples (e.g., sharing dinner with a roommate, helping a younger sibling). Each evening they were asked to think about and write down three good things they could do the next day for another person (or a group of people) and describe them in more detail. The next day they were asked to reflect on whether they had succeeded in the achievement of these goals and write down their related emotions and reflections.

#### Childhood Memories (Placebo Control Condition)

First, the students received a presentation explaining childhood memories with examples (e.g., playing with peers, watching cartoons). Each day they were asked to recall and write down three early childhood memories as accurately as possible.

### Measures

Available Polish versions of the instruments were used. The reliability of each measure is reported in Table 1.

**Table 1:** Descriptive statistics and reliability of measures in the experimental and control groups at four measurement times.

**Variable**	**Measurement**											
	**T0**			**T1**			**T2**			**T3**		
**Group**	***M***	***SD***	**α**	***M***	***SD***	**α**	***M***	***SD***	**α**	***M***	***SD***	**α**
**Prosocial behavior^a^**												
Experimental	6.20	3.27	0.88	6.93	3.27	0.94	6.88	3.23	0.92	6.93	3.21	0.90
Control	5.96	3.52	0.92	6.67	3.24	0.88	6.61	3.27	0.91	6.96	2.89	0.88
**Positive affect**												
Experimental	3.08	0.69	0.85	3.30	0.73	0.91	3.21	0.73	0.90	3.21	0.60	0.86
Control	3.14	0.63	0.85	3.25	0.68	0.87	3.27	0.73	0.91	3.29	0.59	0.83
**Negative affect**												
Experimental	2.80	0.75	0.86	2.49	0.78	0.91	2.56	0.88	0.93	2.52	0.77	0.90
Control	2.44	0.73	0.88	2.34	0.65	0.87	2.38	0.79	0.91	2.52	0.83	0.92
**Positive orientation^b^**												
Experimental	−0.04	0.96	0.88, 0.79, 0.81	0.09	0.94	0.92, 0.80, 0.75	−0.03	1.05	0.87, 0.87, 0.78	0.01	0.93	0.81, 0.85,0.84
Control	0.13	1.02	0.87, 0.78, 0.82	0.12	0.96	0.90, 0.83, 0.85	0.18	1.03	0.88, 0.83, 0.89	0.08	0.96	0.88, 0.68, 0.85

*T0, pretest before the intervention; T1, posttest 1 day after the intervention; T2, follow-up 2 weeks after the intervention; T3, follow-up 4 weeks after the intervention*.

*Descriptive statistics are provided for participants who completed all four measurements and were included in further analyses (n = 89, including n_experimental_ = 41, n_control_ = 48)*.

a *The Guttman split-half coefficient was applied to indicate the reliability of the Social Value Orientation measure*.

b *For positive orientation, the reliabilities of the three measures of its components are reported in the following order: self-esteem, optimism, and life satisfaction*.

Prosocial behavior was assessed with a measure of Social Value Orientation (Van Lange et al., 1997; Polish version: Laguna et al., 2020b), a nine-item decomposed game. The game instructions stated that the participant and the other person who they did know would make choices. Outcomes were presented in terms of points. An example item was the choice among three options: A, 500 points for self and 100 for other; B, 500 points for self and 500 for other; and C, 550 points for self and 300 for other. Only option B represented a prosocial choice for which one point was given (the remaining choices = 0 points); total scores ranged from 0 to 9.

Positive and negative affect was measured using the Positive and Negative Affect Schedule (Watson et al., 1988; Polish version: Fajkowska and Marszał-Wiśniewska, 2009). Participants rated to what extent they had felt 10 types of positive affect (e.g., active, excited) and 10 types of negative affect (e.g., upset, nervous) on a 5-point scale (1 = *very slightly or not at all*, 5 = *extremely*) in the past week.

Positive orientation was evaluated by means of three scales measuring three components of positive orientation based on respondents' experiences in the past week. The 10 items of Rosenberg's (1989; Polish version: Łaguna et al., 2007). Self-Esteem Scale (e.g., “I feel that I have a number of good qualities”) were rated on a 4-point scale (1 = *strongly disagree*, 4 = *strongly agree*). The 10 items of the Life Orientation Test (Scheier et al., 1994; Polish version: Poprawa and Juczyński, 2009), measuring optimism (e.g., “I rarely count on good things happening to me”), were rated on a 5-point scale (1 = *strongly disagree*, 5 = *strongly agree*). Finally, the five items of the Satisfaction With Life Scale (Diener et al., 1985; Polish version: Juczyński, 2001), measuring life satisfaction (e.g., “In most ways, my life is close to my ideal”), were rated on a 7-point scale (1 = *strongly disagree*, 7 = *strongly agree*). Based on the scores on these three scales, we computed the score on the higher order factor of positive orientation resulting from the exploratory factor analysis. A single factor explained, respectively, 41.26 and 41.89% of the variance in the experimental and control groups at all measurement times; factor loadings ranged from 0.41 to 0.84.

## Results

### Dropout Analysis

We tested if the participants who took part in more than one measurement differed from those who took part only in the pretest in age, sex and all study variables. The results showed no statistically significant differences (all *p* > 0.05). There was also no differential dropout rate between the experimental and control groups [$\chi_{\left(1,186\right)}^{2}$ = 2.39, *p* = 0.122]. This demonstrates that noticeable sample size reduction did not cause selection bias.

### Randomization Check

We compared the experimental and control groups on demographics and on the baseline levels of all study variables (pre-test). No statistically significant differences were observed (all *p* > 0.05), which confirmed the effectiveness of randomization.

### Effectiveness of the Intervention

Means and standard deviations for participants who completed four measurements (*n* = 89: *n*_experimental_ = 41, *n*_control_ = 48) and were included in analyses are given in Table 1. Such a sample size allows for detecting relatively large effects (effect size *f* = 0.45, α = 0.05, power = 0.95). The participants followed the daily instructions, performing the assigned tasks for 6–7 days on average (*M*_experimental_ = 6.56, *SD* = 1.05; *M*_control_ = 6.33, *SD* = 1.10).

**Figure 2:**
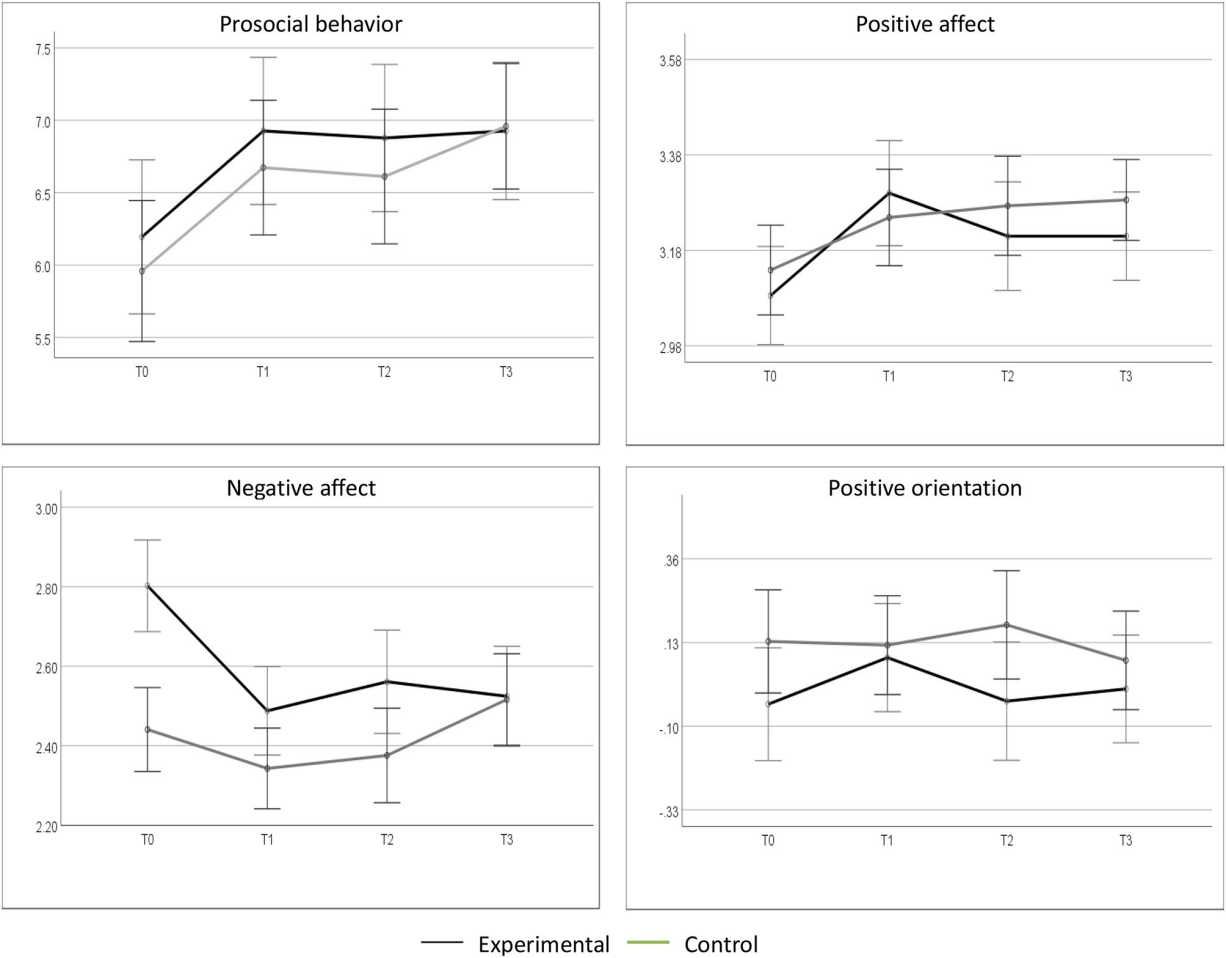
Changes in the mean level (and standard deviations) of prosocial behavior, positive affect, negative affect, and positive orientation in the experimental and control groups at four measurement times.

To test the effectiveness of the intervention, we applied repeated measures ANOVA for each variable of interest. We performed 2 × 4 analyses with Group (experimental vs. control) as the between-subjects factor and Time (T0, T1, T2, T3) as the within-subjects factor. The results (Table 2) revealed statistically significant effects of Time (i.e., changes observed along measurements; Figure 2) on prosocial behavior (η^2^ = 0.07) and on positive (η^2^ = 0.04) and negative affect (η^2^ = 0.03), but not on positive orientation. These effects were nevertheless small; they are explained in details in the next paragraphs. Neither the effect of Group nor the effect of Time × Group interaction turned out to be significant for any of the variables, which means, no between-group differences were detected.

**Table 2:** Repeated measures ANOVA results.

**Dependent variable**	**Time (*****df*** **=** **3, 264)**			**Group (*****df*** **=** **1, 88)**			**Time** ** × ** **Group (*****df*** **=** **3, 264)**			**Contrasts^a^**
	***F***	**η^2^**	***p***	***F***	**η^2^**	***p***	***F***	**η^2^**	***p***	
Prosocial behavior	7.24	0.07	0.001	0.80	0.01	0.778	0.25	0.01	0.844	Con: T0–T1, T0–T3
Positive affect	3.15	0.04	0.030	0.08	0.01	0.775	0.51	0.01	0.659	Exp: T0–T1
Negative affect	2.70	0.03	0.054	1.68	0.02	0.198	1.85	0.02	0.147	Exp: T0–T1
Positive orientation	0.66	0.01	0.555	0.39	0.01	0.535	1.27	0.01	0.286	-

*Con, placebo control group; Exp, experimental group; T0–T1, pretest and posttest comparison; T0–T3, pretest and follow-up 4 weeks after the intervention comparison*.

a *Statistically significant planned contrasts of at least p < 0.05 are reported from the between-group ANOVA (Time × Group), as indicated by Bonferroni tests*.

We based the interpretation of findings on planned contrasts (Gander et al., 2013), evaluated applying Bonferroni test. The scores at every measurement time were compared with pretest. The results revealed statistically significant increases in prosocial behavior in the placebo control group (between *M*_T0_ = 5.96 and *M*_T1_ = 6.67, *p* = 0.041, and between *M*_T0_ = 5.96 and *M*_T3_ = 6.96, *p* = 0.011), but not in the experimental group. As no increases were detected in the experimental group, our expectations concerning the effects of the intervention on prosocial behavior were not confirmed.

Planned contrasts were also applied to test the effects of the intervention on other variables. We detected a significant increase in positive affect (*M*_T0_ = 3.09 < *M*_T1_ = 3.30, *p* = 0.020) and a significant decrease in negative affect (*M*_T0_ = 2.80 > *M*_T1_ = 2.49, *p* = 0.002) in the experimental group a day after the intervention (Figure 2). There were, however, no changes in affect at follow-ups, as compared to pretest. We observed no significant changes in the level of positive orientation.

### Additional Analyses

As the fact that the participants did or did not do the daily tasks may have affected our results, we performed additional analyses. We conducted repeated measures ANOVA with an additional between-subjects factor—namely, the number of daily tasks done. The results show that the number of daily tasks completed has no effect on any of the outcome measures (all *p*s > 0.05).

As one may hypothesize a mediation effect, namely that engagement in prosocial behavior encouraged by the intervention may stimulate affect and beliefs, which in turn may influence prosocial behavior, we performed additional mediation analyses. We applied the macro PROCESS (Hayes, 2013) to test models with prosocial behavior just after the intervention (at T1) as a predictor, positive and negative affect and positive orientation measured after 2 weeks (at T2) as mediators, and prosocial behavior 4 weeks after the intervention (at T3) as a dependent variable. None of these models revealed a statistically significant mediation effect, as all confidence intervals included zero ([−0.02,0.01] for positive affect, [−0.005,0.01] for negative affect, and [−0.10,0.08] for positive orientation). This means that no mediation effects were found.

## Discussion

Our aim was to test the effectiveness of the “three good things for others” intervention, inspired by positive psychology interventions (Seligman et al., 2005; Gander et al., 2018). This short, 1-week online intervention was designed for young adults. In order to test its effectiveness, we carefully planned a randomized controlled trial with four measurements (pretest, posttest, and two follow-ups: 2 and 4 weeks after the intervention) and random assignment of participants to experimental and control groups. The study was a placebo-controlled experiment, in which participants in the control group were engaged in an activity of the same structure and timing. We ensured that randomization was successful and that the attrition of participants did not affect the generalizability of the findings. Except of prosocial behavior we tested other potential outcomes, including positive and negative affect, and positive orientation.

Our study detected some effects of the intervention; however, no increase in prosocial behavior was observed. The results showed a statistically significant increase in prosocial value orientation (Van Lange et al., 1997)—unexpectedly, in the placebo control group but not in the experimental group. Young people prompted to reflect on their childhood memories boosted their prosocial choices. Recalling childhood memories may bring to mind an innocent and morally pure concept of self. Accessing such a sense of heightened morality, people may behave more prosocially (Gino and Desai, 2012). This mechanism may have become activated by the control condition, resulting in a higher level of prosocial behavior. Our results thus demonstrate that prosocial value orientation is malleable; it does not change as a result of the “three good things for others” intervention, however.

The results concerning affect demonstrated a statistically significant increase in positive affect and decrease in negative affect in the experimental group a day after the intervention. No significant changes were observed in the placebo control group. Yet, these effects did not last as long as the next 2 or 4 weeks. This suggests that it is possible to reduce negative affect and to stimulate positive affect using the “three good things for others” intervention, at least for a short time. This result is consistent with other findings concerning positive psychology interventions (Bolier et al., 2013) and prosocial interventions (Schonert-Reichl et al., 2015).

The results concerning positive orientation demonstrated its relative stability, which is in line with findings showing its heritability (Caprara et al., 2009) rather than with those suggesting that it is a state-like phenomenon (Alessandri et al., 2014). Even if other positive psychology interventions operate through the mechanism of elicitation of positive thoughts (Gander et al., 2018), this new intervention did not lead to changes in positive beliefs.

The results of our study demonstrate that it is not easy to stimulate prosocial behavior expressed in cooperative decisions (Van Lange et al., 1997; Balliet et al., 2009) using a short and simple online exercise that involves planning prosocial activities for the following day and then reflecting on the accomplishment of these activities. The exercise did nevertheless result in an immediate increase in positive affect and decrease in negative affect. There are several issues and limitations to consider when looking for these results. First, the intervention makes people aware that their good intentions of helping, sharing, caring, or comforting others (Alessandri et al., 2009; Caprara et al., 2012) were not always actually fulfilled and that they are personally accountable for their actions (as opposed to simply recalling childhood memories). Moreover, although beneficial for others, prosocial behavior requires effort and not always brings gratitude. Second, our exercise was not rooted in considerations concerning the moral significance of prosocial acts or personal values. It can be expected that practices embedded in a religious context (e.g., Thibodeaux, 2014) or moral reasoning may bring more sustainable effects (Łowicki and Zajenkowski, 2019). Third, at least some of prosocial interventions increased prosocial behavior when delivered to children and adolescents (Mesurado et al., 2019; Laguna et al., 2020c). This suggests that it may be easier to stimulate prosocial actions in younger groups, and it is worth testing the effectiveness of our intervention in children. Fourth, our sample was dominated by women who reported a relatively high level of prosocial behavior even before the intervention and who completed the measures at all measurement times. This limits the generalizability of our findings. We observed a noticeable sample size reduction, although drop-out analysis showed that it did not cause selection bias. Our final sample size, similar to other that in studies on the effectiveness of interventions (e.g., Konrath et al., 2015), was relatively small, which may have diminished the ability to detect statistically significant differences between groups. Fifth, we used the decomposed game to measure prosocial behavior (Van Lange et al., 1997; Balliet et al., 2009), as being less prone to social desirability bias than self-reports. Self-report measures may nevertheless be more likely to detect changes (Laguna and Alessandri, 2020), and the way of measuring prosocial behavior may influence the results (Böckler et al., 2018; Laguna et al., 2020c).

Carefully planned in accordance with recent recommendations (Laguna et al., 2020c), our study yielded results that justify recommending the “three good things for others” intervention as a tool stimulating affect but not prosocial behavior or positive beliefs. Further endeavors are therefore needed to develop new interventions that would be more successful in encouraging prosocial actions. Our results raise questions concerning the mechanisms of increasing prosocial behavior and methods of measuring its changes.

## Data Availability Statement

The original contributions presented in the study are included in the article/Supplementary Material, further inquiries can be directed to the corresponding author.

## Ethics Statement

The studies involving human participants were reviewed and approved by Ethics Committee of The John Paul II Catholic University of Lublin, Institute of Psychology. The patients/participants provided their written informed consent to participate in this study.

## Author Contributions

ML contributed to study design. ML and MK contributed to data analysis. All authors contributed to the development of the intervention, collection of the data, writing, and revising the paper.

## Conflict of Interest

The authors declare that the research was conducted in the absence of any commercial or financial relationships that could be construed as a potential conflict of interest.

## Supplementary Material

Supplementary Table Database_Three good things for others.

## References

[B1] Alessandri G. Caprara G. V. Eisenberg N. Steca P. (2009). Reciprocal relations among self-efficacy beliefs and prosociality across time. J. Pers. 77, 1229–1259. 10.1111/j.1467-6494.2009.00580.x 19558437 PMC2771548

[B2] Alessandri G. Zuffianò A. Fabes R. Vecchione M. Martin C. (2014). Linking positive affect and positive self-beliefs in daily life. J. Happiness Stud. 15, 1479–1493. 10.1007/s10902-013-9487-y

[B3] Balliet D. Parks C. Joireman J. (2009). Social value orientation and cooperation in social dilemmas: a meta-analysis. Group Process. Intergroup Relat. 12, 533–547. 10.1177/1368430209105040

[B4] Böckler A. Tusche A. Schmidt P. Singer T. (2018). Distinct mental trainings differentially affect altruistically motivated, norm motivated, and self-reported prosocial behaviour. Sci. Rep. 8:13560. 10.1038/s41598-018-31813-8 30202029 PMC6131389

[B5] Böckler A. Tusche A. Singer T. (2016). The structure of human prosociality: differentiating altruistically motivated, norm motivated, strategically motivated, and self-reported prosocial behavior. Soc. Psychol. Pers. Sci. 7, 530–541. 10.1177/1948550616639650

[B6] Bolier L. Haverman M. Westerhof G. J. Riper H. Smit F. Bohlmeijer E. (2013). Positive psychology interventions: a meta-analysis of randomized controlled studies. BMC Public Health 13:119. 10.1186/1471-2458-13-119 23390882 PMC3599475

[B7] Caprara G. V. Alessandri G. Caprara M. (2019). Associations of positive orientation with health and psychosocial adaptation: a review of findings and perspectives. Asian J. Soc. Psychol. 22, 126–132. 10.1111/ajsp.12325

[B8] Caprara G. V. Alessandri G. Eisenberg N. (2012). Prosociality: the contribution of traits, values, and self-efficacy beliefs. J. Pers. Soc. Psychol. 102, 1289–1303. 10.1037/a0025626 21942280

[B9] Caprara G. V. Fagnani C. Alessandri G. Steca P. Gigantesco A. Sforza L. L. C.. (2009). Human optimal functioning: the genetics of positive orientation towards self, life, and the future. Behav. Genet. 39, 277–284. 10.1007/s10519-009-9267-y 19360463

[B10] Carver C. S. (2005). Emotion theory is about more than affect and cognition: taking triggers and actions into account. Behav. Brain Sci. 28, 198–199. 10.1017/S0140525X05260041

[B11] Carver C. S. (2006). Approach, avoidance, and the self-regulation of affect and action. Motiv. Emot. 30, 105–110. 10.1007/s11031-006-9044-731492567

[B12] Diener E. Emmons R. A. Larsen R. J. Griffin S. (1985). The satisfaction with life scale. J. Pers. Assess. 49, 71–75. 10.1207/s15327752jpa4901_13 16367493

[B13] Donald J. N. Sahdra B. K. Zanden B. V. Duineveld J. J. Atkins P. W. B. Marshall S. L.. (2019). Does your mindfulness benefit others? a systematic review and meta-analysis of the link between mindfulness and prosocial behaviour. Br. J. Psychol. 110, 101–125. 10.1111/bjop.12338 30094812

[B14] Drazkowski D. Kaczmarek L. D. Kashdan T. B. (2017). Gratitude pays: a weekly gratitude intervention influences monetary decisions, physiological responses, and emotional experiences during a trust-related social interaction. Pers. Individ. Dif. 110, 148–153. 10.1016/j.paid.2017.01.043

[B15] Eisenberg N. Fabes R. A. Spinrad T. L. (2007). Prosocial development, in Handbook of Child Psychology, eds Damon W. Lerner R. M. (Hoboken, NJ: John Wiley and Sons, Inc), 646–718.

[B16] Fajkowska M. Marszał-Wiśniewska M. (2009). Właściwości psychometryczne skali pozytywnego i negatywnego afektu—Wersja rozszerzona (PANAS-X). Wstepne wyniki badań w polskiej próbie. Psychol. Rev. 52, 355–387.

[B17] Fredrickson B. L. (2001). The role of positive emotions in positive psychology: the broaden-and-build theory of positive emotions. Am. Psychol. 56:218. 10.1037/0003-066X.56.3.218 11315248 PMC3122271

[B18] Gander F. Proyer R. T. Ruch W. (2018). A placebo-controlled online study on potential mediators of a pleasure-based positive psychology intervention: the role of emotional and cognitive components. J. Happiness Stud. 19, 2035–2048. 10.1007/s10902-017-9909-3

[B19] Gander F. Proyer R. T. Ruch W. Wyss T. (2013). Strength-based positive interventions: further evidence for their potential in enhancing well-being and alleviating depression. J. Happiness Stud. 14, 1241–1259. 10.1007/s10902-012-9380-0

[B20] Gino F. Desai S. D. (2012). Memory lane and morality: how childhood memories promote prosocial behavior. J. Pers. Soc. Psychol. 102, 743–758. 10.1037/a0026565 22181000

[B21] Hayes A. F. (2013). Introduction to Mediation, Moderation, and Conditional Process Analysis. New York, NY: The Guilford Press.

[B22] Hendriks T. Schotanus-Dijkstra M. Hassankhan A. de Jong J. Bohlmeijer E. (2020). The efficacy of multi-component positive psychology interventions: a systematic review and meta-analysis of randomized controlled trials. J. Happiness Stud. 21, 357–390. 10.1007/s10902-019-00082-1

[B23] Juczyński Z. (2001). Skala Satysfakcji z Zycia, in Narzedzia pomiaru w promocji i psychologii zdrowia, ed Juczyíski Z. (Warsaw: Pracownia Testów Psychologicznych Polskiego Towarzystwa Psychologicznego), 134–138.

[B24] Konrath S. Falk E. Fuhrel-Forbis A. Liu M. Swain J. Tolman R.. (2015). Can text messages increase empathy and prosocial behavior? the development and initial validation of text to connect. PLoS ONE 10:e0137585. 10.1371/journal.pone.0137585 26356504 PMC4565638

[B25] Laguna M. (2019). Towards explaining the “how” of positive orientation: the beliefs-affect-engagement model. Asian J. Soc. Psychol. 22, 133–139. 10.1111/ajsp.12336

[B26] Laguna M. Alessandri G. (2020). Personal resources and prosocial goal realization: The interplay of traits and states. Submited.

[B27] Laguna M. De Longis E. Mazur Z. Alessandri G. (2020a). Explaining prosocial behavior from the between- and within-person perspectives: a role of positive orientation and positive affect. Submited.

[B28] Laguna M. Kedra M. Mazur Z. (2020b). The effectiveness of the prosocial goals intervention: a randomized controlled trial. Submitted.

[B29] Łaguna M. Lachowicz-Tabaczek K. Dzwonkowska I. (2007). Skala Samooceny SES Morrisa Rosenberga—Polska adaptacja metody. Soc. Psychol. 2, 164–176.

[B30] Laguna M. Mazur Z. Kedra M. Ostrowski K. (2020c). Interventions stimulating prosocial helping behavior: a systematic review. J. Appl. Soc. Psychol. 50, 676–696. 10.1111/jasp.12704

[B31] Łowicki P. Zajenkowski M. (2019). Religiousness is associated with higher empathic concern—evidence from self- and other-ratings. Psychol. Relig. Spiritual. 10.1037/rel0000299

[B32] Meier S. (2007). A survey of economic theories and field evidence on pro-social behavior, in Economics and Psychology: A Promising New Cross-Disciplinary Field, eds Frey B. S. Stutzer A. (Cambridge, MA: MIT Press), 51–87.

[B33] Mesurado B. Guerra P. Richaud M. C. Rodriguez L. M. (2019). Effectiveness of prosocial behavior interventions: a meta-analysis, in Psychiatry and Neuroscience Update: From Translational Research to a Humanistic Approach—Vol. III, eds Gargiulo Á P. Mesones Arroyo H. L. (New York, NY: Springer International Publishing), 259–271.

[B34] Moore S. Diener E. Tan K. (2018). Using multiple methods to more fully understand causal relations: positive affect enhances social relationships, in Handbook of Well-Being, eds Diener E. Oishi S. Tay L. (Salt Lake City, UT: Noba Scholar), 1–17.

[B35] Plemmons S. A. Weiss H. M. (2013). Goals and affect, in New Developments in Goal Setting and Task Performance, eds Locke E. A. Latham G. P. (London: Routledge), 117–132.

[B36] Poprawa R. Juczyński Z. (2009). Adaptacja Testu Orientacji Zyciowej LOT-R, in Narzedzia pomiaru w promocji i psychologii zdrowia, 2nd edn, ed Juczyński Z. (Warsaw: Pracownia Testów Psychologicznych Polskiego Towarzystwa Psychologicznego).

[B37] Rosenberg M. (1989). Society and the Adolescent Self-Image, Rev. Edn. Middletown, CT: Wesleyan University Press.

[B38] Scheier M. F. Carver C. S. Bridges M. W. (1994). Distinguishing optimism from neuroticism (and trait anxiety, self-mastery, and self-esteem): a reevaluation of the life orientation test. J. Pers. Soc. Psychol. 67, 1063–1078. 10.1037/0022-3514.67.6.1063 7815302

[B39] Schonert-Reichl K. A. Oberle E. Lawlor M. S. Abbott D. Thomson K. Oberlander T. F.. (2015). Enhancing cognitive and social-emotional development through a simple-to-administer mindfulness-based school program for elementary school children: a randomized controlled trial. Dev. Psychol. 51, 52–66. 10.1037/a0038454 25546595 PMC4323355

[B40] Seligman M. E. P. Steen T. A. Park N. Peterson C. (2005). Positive psychology progress: empirical validation of interventions. Am. Psychol. 60, 410–421. 10.1037/0003-066X.60.5.410 16045394

[B41] Sin N. L. Lyubomirsky S. (2009). Enhancing well-being and alleviating depressive symptoms with positive psychology interventions: a practice-friendly meta-analysis. J. Clin. Psychol. 65, 467–487. 10.1002/jclp.20593 19301241

[B42] Thibodeaux M. E. (2014). Reimagining the Ignatian Examen: Fresh Ways to Pray From Your Day. Chicago, IL: Loyola Press.

[B43] Thoits P. A. Hewitt L. N. (2001). Volunteer work and well-being. J. Health Soc. Behav. 42, 115–131. 10.2307/3090173 11467248

[B44] Van Lange P. A. Otten W. De Bruin E. M. Joireman J. A. (1997). Development of prosocial, individualistic, and competitive orientations: theory and preliminary evidence. J. Pers. Soc. Psychol. 73, 733–746. 10.1037/0022-3514.73.4.733 9325591

[B45] Walton G. M. (2014). The new science of wise psychological interventions. Curr. Dir. Psychol. Sci. 23, 73–82. 10.1177/0963721413512856

[B46] Watson D. Clark L. A. Tellegen A. (1988). Development and validation of brief measures of positive and negative affect: the PANAS scales. J. Pers. Soc. Psychol. 54, 1063–1070. 10.1037/0022-3514.54.6.1063 3397865

